# Positive Selection Driving Cytoplasmic Genome Evolution of the Medicinally Important Ginseng Plant Genus Panax

**DOI:** 10.3389/fpls.2018.00359

**Published:** 2018-04-04

**Authors:** Peng Jiang, Feng-Xue Shi, Ming-Rui Li, Bao Liu, Jun Wen, Hong-Xing Xiao, Lin-Feng Li

**Affiliations:** ^1^Key Laboratory of Molecular Epigenetics of the Ministry of Education, Northeast Normal University, Changchun, China; ^2^Northeast Normal University Natural History Museum, Changchun, China; ^3^Ministry of Education Key Laboratory for Biodiversity Science and Ecological Engineering, School of Life Sciences, Fudan University, Shanghai, China; ^4^Department of Botany, National Museum of Natural History, Smithsonian Institution, Washington, DC, United States

**Keywords:** Araliaceae, cytoplasmic genome, genome sequencing, *Panax*, positive selection

## Abstract

*Panax* L. (the ginseng genus) is a shade-demanding group within the family Araliaceae and all of its species are of crucial significance in traditional Chinese medicine. Phylogenetic and biogeographic analyses demonstrated that two rounds of whole genome duplications accompanying with geographic and ecological isolations promoted the diversification of *Panax* species. However, contributions of the cytoplasmic genomes to the adaptive evolution of *Panax* species remained largely uninvestigated. In this study, we sequenced the chloroplast and mitochondrial genomes of 11 accessions belonging to seven *Panax* species. Our results show that heterogeneity in nucleotide substitution rate is abundant in both of the two cytoplasmic genomes, with the mitochondrial genome possessing more variants at the total level but the chloroplast showing higher sequence polymorphisms at the genic regions. Genome-wide scanning of positive selection identified five and 12 genes from the chloroplast and mitochondrial genomes, respectively. Functional analyses further revealed that these selected genes play important roles in plant development, cellular metabolism and adaptation. We therefore conclude that positive selection might be one of the potential evolutionary forces that shaped nucleotide variation pattern of these *Panax* species. In particular, the mitochondrial genes evolved under stronger selective pressure compared to the chloroplast genes.

## Introduction

Flowering plants contain three genomes in distinct compartments, namely nuclear, mitochondrion and plastid, with each possessing different evolutionary trajectories and relatively independent genetic systems (Smith and Keeling, 2015). This tripartite distribution of nuclear and cytoplasmic genomes has profound impacts on the evolution and diversification of flowering plants (Roux et al., 2016; Sharbrough et al., 2017). For example, genes showing interaction between nuclear and organellar compartments are thought to be co-adapted in higher plants (Kleine et al., 2009; Greiner and Bock, 2013), in particular to those species that have undergone multiple rounds of whole genome duplication (WGD) (Sloan et al., 2013; Gong et al., 2014; Sharbrough et al., 2017). Roles of nuclear genome in the adaptation and diversification of higher plants have been extensively investigated; examples of such studies include how the changes in genome architecture responding to hybridization and polyploidy (Abbott et al., 2013; Soltis et al., 2015; Wendel et al., 2016; Sherman-Broyles et al., 2017; Spoelhof et al., 2017) as well as the molecular basis underlying the Evo-Devo process (De Bruijn et al., 2012; Fernández-Mazuecos and Glover, 2017). In contrast, contributions of the cytoplasmic genomes of flowering plants to local adaptation and long-term evolutionary significance are less studied (reviewed in Bock et al., 2014).

In flowering plants, cytoplasmic genome consists of chloroplast and mitochondrial genomes, which evolved separately from once free-living bacteria by endosymbiosis (Gray, 1992; Zimorski et al., 2014), but now are subcellular bioenergetics organelles with their own genetic systems (Allen, 2015). Mitochondrion is the first cytoplasmic genome captured by eukaryotes (Hjort et al., 2010), which produces energy by oxidative phosphorylation and channeling electrons through the respiratory chain complexes (Björkholm et al., 2015). While the mitochondrial genome of flowering plants shows very low rate of nucleotide substitution, substantial variations in genome size and structure are observed, such as structural rearrangements, massive gene loss, and frequent endogenous and foreign gene transfer (reviewed in Chen et al., 2017a). For example, the parasitic species *Viscum scurruloideum* possesses a mitochondrial genome with only ~65.9 kb in size, but *Silene conica* contains a much larger mitochondrial genome (~11.3 Mb in size) (Sloan et al., 2012; Skippington et al., 2015). In contrast, the chloroplast genome of flowering plants is relatively conserved at both structure and gene content levels (Yagi and Shiina, 2014; Smith and Keeling, 2015). Most flowering plants have a conserved quadripartite structure in the chloroplast genome of ~100-220 kb in size, which consists of a large single copy (LSC), a small single copy (SSC), and a pair of large inverted repeats (IRA and IRB) (Turmel et al., 2002; Wicke et al., 2011; Daniell et al., 2016). In addition to the photosynthesis that converts solar energy to carbohydrates and oxygen, chloroplast genome also plays crucial roles in plant growth and development, including nitrate and sulfate assimilation, amino acids, chlorophyll and carotenoids biosynthesis (Jensen and Leister, 2014; Daniell et al., 2016).

Compared to the nuclear genome that natural selection is one of the major evolutionary forces maintaining genetic variations at both intra- and inter-species levels, sequence polymorphisms existed in cytoplasmic genomes have long been thought as selectively neutral in the evolutionary process of flowering plants (reviewed in Bock et al., 2014). However, increasing evidence from recent genetic studies tends to challenge the neutral evolution assumption by showing that DNA polymorphisms are frequently adaptive in both mitochondrial and chloroplast genomes (reviewed in Bock et al., 2014). Evidence in support of this hypothesis came from the observations that a series of mitochondrial and chloroplast genes were found to show accelerated rates of nucleotide substitution. For example, the self-replication genes (e.g., *rpo, rpl*, and *rps* genes) of the chloroplast genome also exhibit relatively high nucleotide substitution rate compared to other genes (Guisinger et al., 2008; Weng et al., 2012). Additional evidence that indicates non-neutral evolution of cytoplasmic genome is reported in the chloroplast protein Rubisco in which the plastid-encoded large subunit (*rbcL*) not only shows signature of selection in most land plant species (reviewed in Bock et al., 2014), but also has profound effects on the concerted evolution of the nuclear-encoded small subunit (*rbcS*) in polyploid species (Gong et al., 2012, 2014). These empirical results apparently suggest that while parts of cytoplasmic genomes may evolve neutrally, some specific genes are frequently co-adapted with nuclear genes.

*Panax* L. (the ginseng genus) is a medicinally important genus within the family Araliaceae and all the approximately eight species are also of cultural significance for traditional Chinese medicine (Choi and Wen, 2000; Wen, 2001a,b; Zuo et al., 2011, 2015; Li and Wen, 2016). Phylogenetic analyses based on cytoplasmic and nuclear genes revealed two independent origins of the East Asian and North American disjunct distribution in *Panax*, with the diploid species *P. trifolius* and one of the tetraploid species *P. quinquefolius* distributed in eastern North America and the remaining species inhabiting in both cold temperate and warm subtropical and tropical regions of East Asia (Wen and Zimmer, 1996; Lee and Wen, 2004; Shi et al., 2015; Zuo et al., 2017). With regard to the mechanisms underlying the species diversification, two rounds of WGD have been hypothesized as the major driving force that caused rapid genomic divergence among *Panax* species (Choi et al., 2013, 2014; Kim et al., 2014; Shi et al., 2015). Then, geographic isolation along with shifting in ecological habitats has further promoted the evolutionary radiation of *Panax* (Shi et al., 2015; Zuo et al., 2017). These attributes make *Panax* an ideal system to investigate how the natural selection accompanying geographic and ecological isolations shape the patterns of nucleotide polymorphism at both intra- and inter-specific levels. In particular, nuclear and cytoplasmic reference genomes of several *Panax* species have been recently sequenced, including *P. ginseng* (Zhao et al., 2015; Jiang et al., 2017), *P. notoginseng* (Zhang et al., 2016, 2017; Chen et al., 2017b) and *P. quinquefolius* (Han et al., 2016). These available genomic resources provide a useful platform to evaluate adaptation and diversification of *Panax* at the genome-scale.

In this study, we sequenced the chloroplast and mitochondrial genomes of three diploid and three tetraploid *Panax* species along with five varieties of the *P. bipinnatifidus* species complex. All these *Panax* species are shade-demanding perennial herbs and inhabit the cool shaded habitats (Liu and Xiao, 1992; Chen et al., 2014, 2016). In the case of the agro-economically important species *P. ginseng*, while cultivated ginseng harbored similar level of nucleotide diversity compared to the wild ginseng in the nuclear genome, obviously lower sequence polymorphisms were found in the chloroplast genome of cultivated ginseng (Li et al., 2015, 2017). Specifically, cytoplasmic genomes as well as the cytoplasmic proteins encoded by the nuclear genome are the targets of selection in the cultivated ginseng during local adaptation and domestication processes (Li et al., 2017). Similarly, the increase in specific leaf area and chlorophyll content of the species *P. notoginseng* is negatively correlated with the growth irradiance level (Chen et al., 2014, 2016). It indicates the possibility that cytoplasmic genomes may not evolve neutrally in *Panax* species. To experimentally test this hypothesis, the current study attempts to evaluate how natural selection acts on the cytoplasmic genomes during the long-term evolutionary process of *Panax*. Our findings may provide new evidence on the non-neutral evolution model of cytoplasmic genome in *Panax*.

## Materials and methods

### Plant materials and DNA extraction

**Figure 1:**
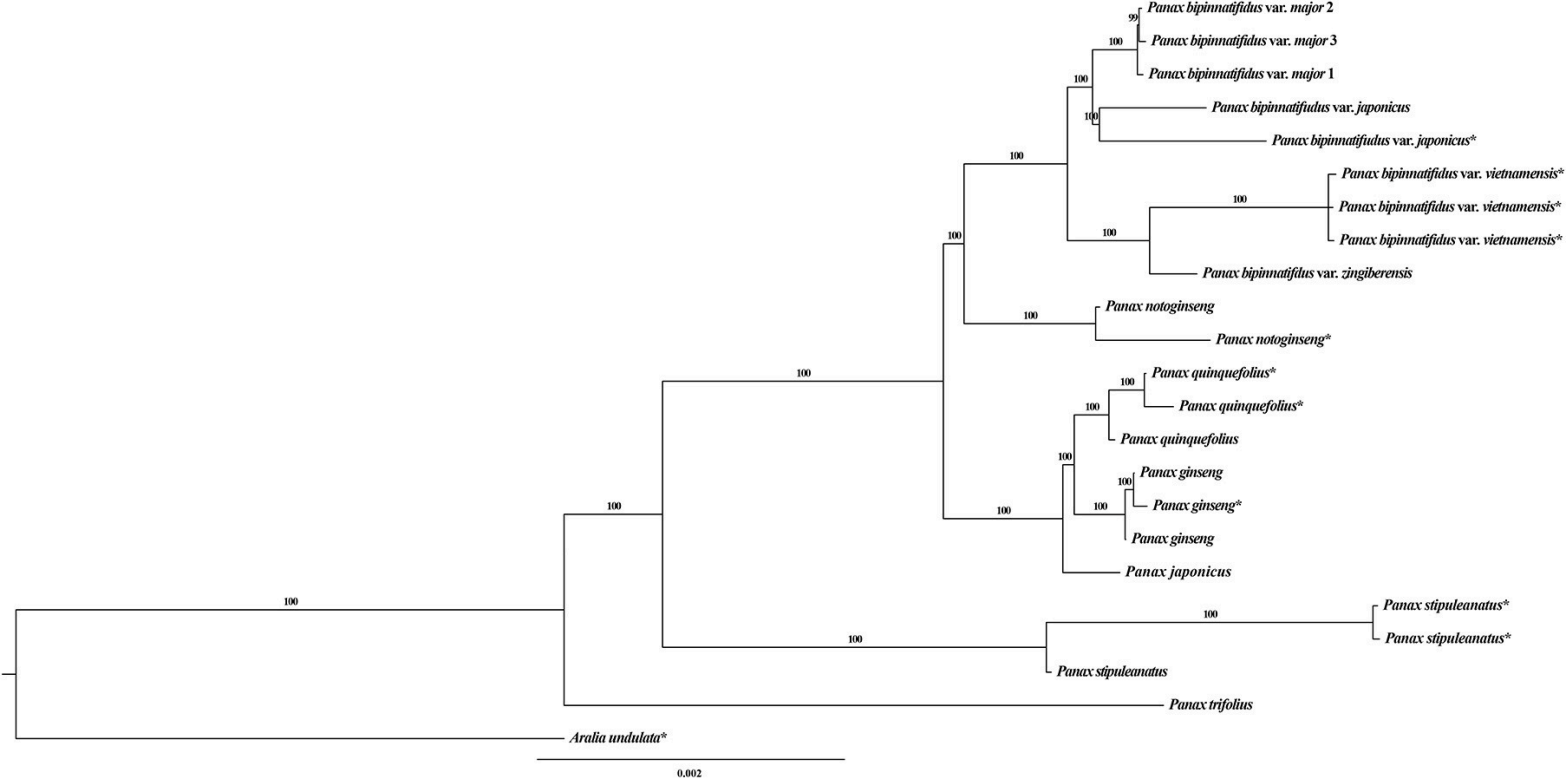
Bayesian tree of the genus Panax based on the whole chloroplast genome. ^*^indicates these sequences were downloaded from GenBank. Numbers on each branch are the posterior probabilities.

In previous studies, Wen and colleagues classified the genus *Panax* as seven well-recognized species and a group of not well-defined species represented by *P. bipinnatifidus* (Wen, 2001a; Zuo et al., 2011, 2015, 2017). Following their classification system, our specimens used in this study covered the *P. bipinnatifidus* species complex and six of the seven *Panax* species (Table S1). To further distinguish the five *P. bipinnatifidus* accessions used, we named them as *P. bipinnatifidus* var. *japonicus, P. bipinnatifidus* var. *major, P. bipinnatifidus* var. *zingiberensis* and *P. bipinnatifidus* var. *vietnamensis*, respectively (Figure 1 and Table S1). The diploid species *P. trifolius* and the tetraploid species *P. japonicus* were obtained from commercial farmer's markets in United States and Japan, respectively, and validated through comparing the DNA sequences of chloroplast *rbcL* gene with the available *Panax* species deposited in GenBank (Supplementary Data 1). The five varieties of *P. bipinnatifidus* species complex and two diploid species, *P. notoginseng* and *P. stipuleanatus*, were kindly provided by our collaborators. The remaining two tetraploid species *P. ginseng* and *P. quinquefolius* were collected in our previous studies (Li et al., 2015; Shi et al., 2015). Genomic DNAs of *P. notoginseng, P. stipuleanatus* and the five varieties of *P. bipinnatifidus* species complex were extracted from the silica-gel dried leaves using TianGen Plant Kit (TianGen, Beijing, China) following the manufacturer's instructions. Genomic DNAs of the remaining five species were extracted from fresh root tissue using TianGen Plant Kit.

### DNA sequencing, assembly and quality control

DNA libraries of the 11 *Panax* accessions were constructed by Novogene (Tianjin, China) with an average insertion size of 350 bp, and sequenced with the Illumina X10 platform (Illumina, Inc., San Diego, California, USA). The quality of obtained raw reads were evaluated for each accession using FASTQC (Schmieder and Edwards, 2011) and the low quality reads (Phred < 30) were removed using NGSQCtoolkit (Patel and Jain, 2012). Filtered reads were mapped to available chloroplast (GenBank accession: KF431956) and mitochondrial (GenBank accession: KF735063) reference genomes of *P. ginseng* using Burrows-Wheeler Aligner (Li and Durbin, 2010) with the default setting. To access the mapping quality of these *Panax* accessions, we also report the read depth for each accession and all *Panax* species using SAMtools (Li et al., 2009) with the parameter “samtools depth.” Single nucleotide polymorphisms (SNPs) and insertion/deletion (INDELs) were determined using SAMtools (Li et al., 2009), with the command “mpileup -ugst DP,SP -C 50 -q 30 -Q 20” and “bcftools call –vc.” Raw variants (SNPs and INDELs) were further filtered using Perl script with the cutoff “mapping quality (MQ) >30, read depth (RD) >3.” The values of DP at reported positions were employed to determine the missing data (DP = 0) for each of the *Panax* accessions. All filtered variants were subjected to subsequent data analyses.

### Phylogenetic analyses

To remove the systematic bias that caused by missing data, only the SNPs that are non-missing in any of the 11 *Panax* accessions were used to construct the phylogenetic tree. Positions with missing data were removed using the software VCFtools (Danecek et al., 2011) with the parameter “vcftools –max-missing 1.” Filtered variant call format (VCF) were converted into FASTA format using Perl scripts. We realized that our data analyses relied on the assumption of no structural variations at both cytoplasmic genomes. To further test the reliability of our datasets, we downloaded 10 available *Panax* chloroplast genomes from GenBank and compared them with our data generated in this study. These chloroplast genome sequences include *P. japonicus* (KP036469), *P. japonicus* var. *bipinnatifidus* (KX46), *P. notoginseng* (KP036468), *P. ginseng* (KM067393), *P. stipuleanatus* (KX247147), two accessions of *P. quinquefolius* (KT028714 and KM088018), and three accessions of *P. bipinnafidus* var. *vietnamensis* (KP036470, KU059178, and KP036471). The closely related species *Aralia undulata* (KC456163) was employed as outgroup (Wen, 2011; Li et al., 2013). For the mitochondrial genome, only the reference species *P. ginseng* is available in GenBank (KF735063). We therefore employed the first diverging species of the genus *P. trifolius* (Wen and Zimmer, 1996; Lee and Wen, 2004; Zuo et al., 2017) to root the phylogenetic tree. In addition, we also employed the genic matrices to reconstruct the NJ tree and to assess the impacts of mitochondrial structural variations on the subsequent data analyses. Nucleotide substitution models of chloroplast and mitochondrial matrices were estimated using jModeltest separately (Darriba et al., 2012). The best-fit models for chloroplast and mitochondrial genomes are TVM+I+G and GTR+I+G, respectively. Bayesian analyses for the two cytoplasmic genomes were performed using MrBayes 3.2.6 (Ronquist et al., 2012), separately. For each of the two data sets, two independent Markov chains were performed with 1,000,000 generations. Divergence of the two independent runs was estimated by stopping the Bayesian iterations when the average standard deviation is below 0.01. Posterior probabilities on each branch were calculated as the majority-rule consensus of all sampled Bayesian trees with the first 25% discarded as burn-in. Topologies of the obtained Bayesian trees were presented using FigTree 1.4.3 (Rambaut, 2009) and modified with the program Adobe Illustrator CS5.

### Nucleotide variation pattern and identification of selected genes

Distributions of sequence polymorphism were evaluated for the newly generated datasets of chloroplast and mitochondrial genomes using VCFtools (Danecek et al., 2011) with the command “vcftools–SNPdensity,” respectively. Numbers of variants were illustrated for all 11 *Panax* accessions with a sliding window size of 300 bp and visualized using R scripts. As very few variants were identified in each *Panax* accession, we visualized the variation distribution pattern with a sliding window size of 1,000 bp using R scripts. Coordination of each cytoplasmic gene was obtained from the available annotations of chloroplast (KF431956) and mitochondrial (KF735063) genomes. Variant distribution for each *Panax* accession was also presented using R scripts. All protein coding genes of the two cytoplasmic genomes were translated into amino acid based on the available annotations. Then, synonymous and non-synonymous substitutions of each gene were calculated by counting the numbers of each type of the variants. To further identify the genes under selection, we scanned the chloroplast and mitochondrial genomes using codelml of the package PAML (Yang, 2007). Only the genic matrices of the two cytoplasmic genomes were employed in the selection analyses. To further eliminate the data bias caused by the inter-genic regions, the chloroplast NJ tree was used to determine the phylogenetic relationships of these *Panax* species. Five site models (M0, M1a, M2a, M7, and M8) were employed to detect the signatures of adaptation across chloroplast and mitochondrial genomes. Likelihood ratio test (LRT) of the comparisons (M1a vs. M2a and M7 vs. M8) were used to evaluate of the selection strength and the *p* value of Chi square smaller than 0.05 is thought as significant. Genes that showed significant selection in both comparisons were defined as selected genes. Then, the Bayes Empirical Bayes (BEB) inference (Yang et al., 2005) was implemented in site models M2a and M8 to estimate the posterior probabilities and positive selection pressures of the selected genes.

## Results

### Sequence polymorphism

Total mapped reads on the chloroplast (156,355 bp) and mitochondrial (464,680 bp) reference genomes of these *Panax* accessions ranged from 140,136,350 (33,838 x coverage) in *P. japonicus* to 14,003,766 in *P. notoginseng* (3,381 x coverage), with an average depth of 14,356 x across the 11 *Panax* accessions (Table 1). All data generated in this study were submitted to GenBank under the bioproject number PRJNA428843. Differences in mapped reads and coverage depth observed in these accessions are mainly due to the total reads obtained for each accession. Given that the lowest genome coverage is ~3,381 x, we think difference in sequencing depth would not affect the accuracy of the subsequent data analyses. This conclusion is also supported by the statistics of read depth that while some mitochondrial regions possess relatively low read depth, most of the two cytoplasmic genomes show high genome coverage across these *Panax* accessions (Figure S1 and Supplementary Data 2). To this end, we calculated the number of variants for each of the 11 *Panax* accessions by comparing them with the reference *P. ginseng*. For the chloroplast genome, our results showed that only one variant was observed between the *P. ginseng* accession and reference sequence, indicating low level of intra-specific polymorphisms of *P. ginseng* (Table 1). Likewise, relatively low sequence polymorphisms were found in the two tetraploid species *P. quinquefolius* (129 SNPs, 0.08% of the chloroplast genome) and *P. japonicus* (115 SNPs, 0.07%) that are phylogenetically very close to the reference species (Table 1; also see Wen and Zimmer, 1996). In contrast, the two diploid species *P. trifolius* (1,242 SNPs, 0.8%) and *P. stipuleanatus* (995 SNPs, 0.6%) that show a large distance to the reference species possess 2–10 times more of the number of variants compared to the other species (Table 1). Similar trends were also observed in the mitochondrial genome where only 100 intra-specific variants (0.02%) were reported in *P. ginseng*, whereas the inter-specific variants varied from 1,009 (0.2%) in *P. japonicus* to 3,370 (0.7%) in *P. notoginseng* (Table 1). Thus, based on comparisons of the two cytoplasmic genomes, our results reveal that the mitochondrial genome harbors more variants at both overall and individual accession levels in comparison with the chloroplast genome.

**Table 1:** Variant distributions of chloroplast and mitochondrial genomes across the 11 *Panax* accessions.

**Species name**	**Mapped reads**	**Coverage depth**	**Chloroplast**		**Mitochondrion**	
			**Total sites**	**Genic sites**	**Total sites**	**Genic sites**
*Panax ginseng*	22,059,601	5,327	1	1	100	1
*Panax quinquefolius*	41,019,204	9,905	129	29	1,259	13
*Panax japonicus*	140,136,350	33,838	115	36	1,009	4
*Panax bipinnatifidus* var*. major* 1	67,923,657	16,401	356	129	1,897	40
*Panax bipinnatifidus* var*. major* 2	54,686,498	13,205	359	129	1,890	39
*Panax bipinnatifidus* var*. major* 3	80,414,250	19,417	351	128	1,969	40
*Panax bipinnatifidus* var. *japonicus*	49,906,798	12,051	436	146	2,735	42
*Panax bipinnatifidus* var*. zingiberensis*	46,347,969	11,191	417	146	1,119	14
*Panax notoginseng*	14,003,766	3,381	418	132	3,370	89
*Panax stipuleanatus*	26,150,000	6,314	995	359	2,504	89
*Panax trifolius*	111,196,691	26,850	1,242	437	2,230	85

While a small number of sequence polymorphisms were identified in the chloroplast genome, a high proportion of these variants were located in protein-coding genes (Table 1). For example, 29 (22.5%) and 36 (31.3%) of the total SNPs were identified at the genic regions of *P. japonicus* and *P. quinquefolius*, respectively. Similarly, all the remaining species also show moderate percentage of variants (31.6–36.5%) at the genic regions. In contrast, while obviously more sequence polymorphisms were found in the mitochondrial genome compared to the chloroplast, only a small portion of these variants was located at the genic regions (Table 1). For example, only one of the 100 SNPs was identified at the genic regions of *P. ginseng*, suggesting that the mitochondrial coding regions are highly conserved within *P. ginseng*. Likewise, only 0.4–3.8% of the total inter-specific variants are identified at genic regions of the mitochondrial genome (Table 1). It should be noted that the tetraploid species *P. japonicus* possesses the lowest inter-specific variants at total sites of both chloroplast and mitochondrial genomes. On the contrary, while the species *P. trifolius* harbors the highest number of inter-specific variants at both genic and total sites in chloroplast genome, the species *P. notoginseng* shows more genic and total variants compared to other *Panax* species. Notably, a variety of the *P. bipinnatifidus* species complex, var. *zingiberensis*, possesses much less sequence polymorphisms at both genic and total sites compared to the other four varieties of the species in the mitochondrial genome (Table 1), suggesting the evolutionary history of mitochondrion is more complicated relative to chloroplast within *Panax*.

### Phylogenetic analysis and variant distribution

The topology of our Bayesian chloroplast tree is largely congruent with that in previously published studies (Shi et al., 2015; Zuo et al., 2017). Briefly, the diploid species *P. trifolius* and *P. stipuleanatus* constitute the two most basally diverged clades and the remaining species fall into two sister clades (Figure 1), with one of which containing the three tetraploid species, (*P. ginseng, P. japonicus* and *P. quinquefolius*), and the other clade including the diploid species *P. notoginseng* and nine accessions of the *P. bipinatifidus* species complex. We noted that chloroplast genome sequences of the accessions *P. japonicus* (KP036469) and *P. japonicus* var. *bipinnatifidus* (KX247146) downloaded from GenBank were mislabeled. We therefore re-named the two accessions as *P. bipinnatifidus* var. *japonicus* (KP036469) and *P. stipuleanatus* (KX247146) according to the topologies of this study and the previous studies (Shi et al., 2015; Zuo et al., 2017). Compared to the chloroplast phylogenetic tree, different topologies were observed in the mitochondrial genome where two varieties of the *P. bipinatifidus* species complex, var. *zingiberensis* and var. *japonicus*, group together with three tetraploid species with high posterior probability values (Figure S2A). As most of the variants were located at the inter-genic regions, high sequence polymorphism might have resulted in the nucleotide mutation saturation. We therefore only employed the variants located at the genic regions to reconstruct the phylogenetic tree of all *Panax* accessions. Topologies of the resulting phylogenetic tree are highly similar to those that are based on the total polymorphic sites, with the three tetraploid species (*P. ginseng, P. quinquefolius*, and *P. japonicus*) and two varieties of *P. bipinatufidus* being grouped together as a polytomy (Figure S2B). These observations are consistent with the hypothesis based on the variation pattern that the evolutionary trajectory of mitochondrial genome is much more complex compared to the chloroplast genome of *Panax*.

**Figure 2:**
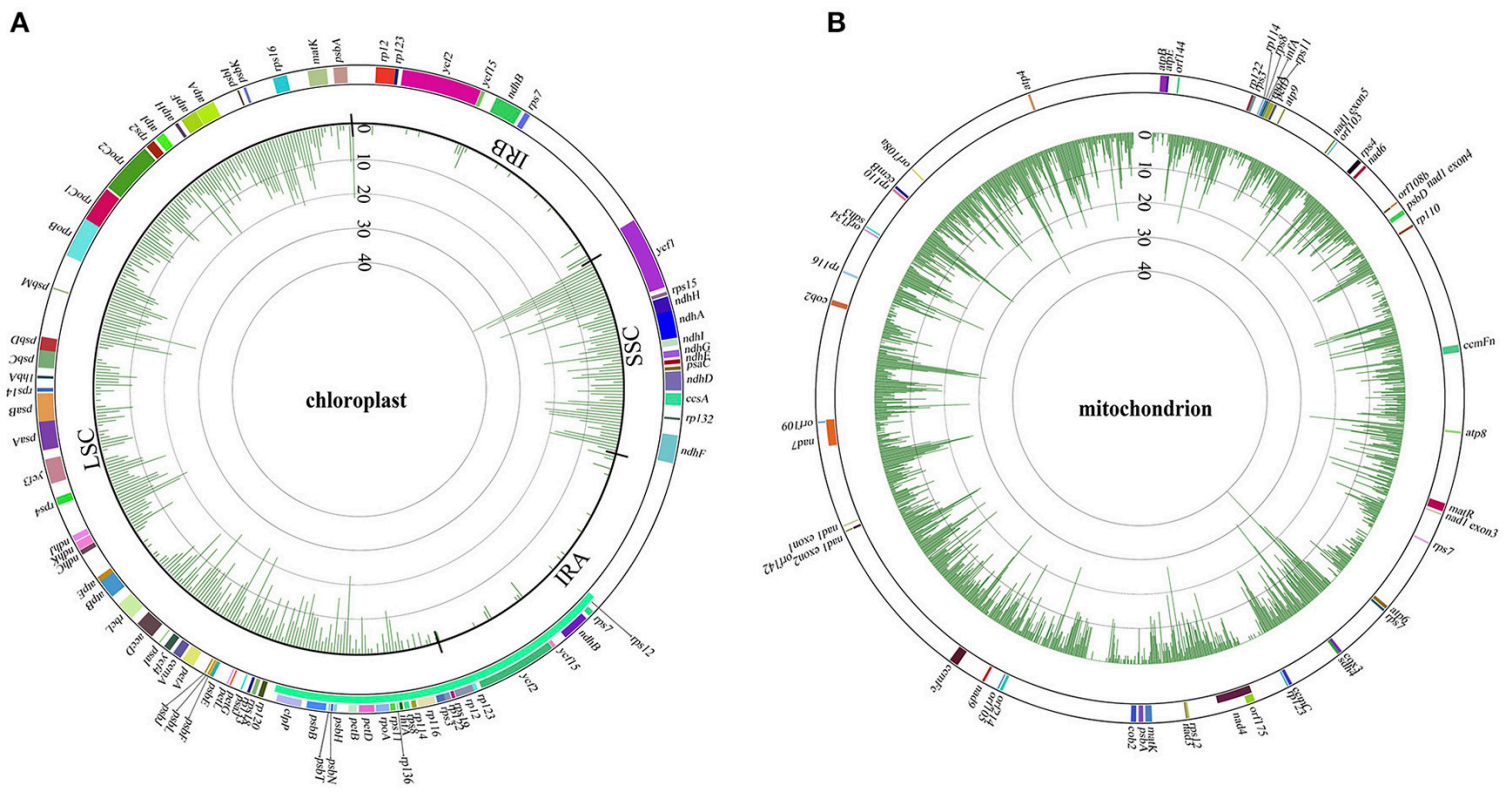
Variant distributions of chloroplast **(A)** and mitochondrial **(B)** genomes in Panax at the overall level. Colors in the outer cycle represent the location and length of each gene in the two cytoplasmic genomes. Numbers in the inner cycles are the total variants within each 300 sliding windows.

With the general characteristics of the sequence polymorphisms and phylogenetic topologies of *Panax* outlined above, we briefly discuss the correlations between phylogenetic relationship and variant distribution for each of these *Panax* accessions below. As expected, the closer the species is related to the reference genome, the fewer variants were observed in both the chloroplast and mitochondrial genomes (Figures S3, S4). At the overall level, both the LSC and SSC harbor relatively higher nucleotide polymorphisms compared to those of the two inverted repeat regions (IRA and IRB) (Figure 2A). A similar phenomenon was also observed at the individual accession level where all the species show obviously low sequence polymorphisms at the IRA and IRB regions (Figure S3). For the mitochondrial genome, while the total number of sequence polymorphism is higher relative to the chloroplast genome, the overall distribution pattern of variants was random across the mitochondrial genome (Figure 2B). In particular, distinct distribution patterns were observed among these *Panax* accessions in the mitochondrial genome (Figure S4).

### Identification of genes under selection

To identify genes under selection in the chloroplast and mitochondrial genomes, we calculated the numbers of synonymous (syn) and non-synonymous (nonsyn) mutations for each gene separately (Tables S2, S3). The syn and nonsyn substitutions were determined by comparing their translated protein sequences with the reference genome. For the chloroplast genome, higher numbers of syn and non-syn substitutions were found in the more basally diverged species *P. trifolius* (syn: 229 and nonsyn: 205) and *P. stipuleanatus* (syn: 166 and nonsyn: 191) compared to the other species (syn: from 18 to 70 and nonsyn: from 10 to 73) that are phylogenetically closer to *P. ginseng*. Specifically, the ratio of syn/nonsyn is greater than or nearly equal to 1 in most of these *Panax* accessions. In the mitochondrial genome, by contrast, all species assessed in this study possess more non-synonymous mutations at the total level. At the individual gene level, sequence polymorphisms are quite different among genes in both chloroplast and mitochondrial genomes (Tables S2, S3). For the chloroplast genome, we found that some genes related to DNA-dependent RNA polymerase (*rpoB, rpoC1*, and *rpoC2*), NADH-dehydrogenase (*ndhD, ndhF*, and *ndhH*), rubisco (*rbcL*) and unknown function (*ycf1*) harbor relatively more variants (Table S2). Likewise, the mitochondrial genes related to ribosomal protein (*rps4*), maturates (*matR*), and ATP synthase (*atp6* and *atp8*) also show relatively high sequence diversity at the genic regions (Table S3). To further identify the genes that evolved under natural selection, we scanned the chloroplast and mitochondrial genomes by comparing the ratio of d_N_/d_S_. Our statistical test on neutralist identified five (*rbcL, clpP, atpF, ycf1*, and *ycf2*) and 12 (*atp6, atp8, matR, nad3, rps4, rps11, sdh3, sdh4, ccmB, ccmC, ccmFc*, and *ccmFn*) genes are under significant positive selection (*p* < 0.05) in the chloroplast and mitochondrial genomes, respectively (Table 2). We noted that these genes under selection are partially overlapped with those of high sequence polymorphism genes. For example, 281 variants (4.9% of the total length) were determined at the *ycf1* gene, 218 of which are nonsyn substitutions across the 11 *Panax* accessions. Most importantly, BEB simulation of the *ycf1* gene identified seven and eight nonsyn sites that are the targets of positive selection in the M2a and M8 models, respectively (Table 2). Likewise, while sequence polymorphisms of the remaining four chloroplast genes were not as high as the *ycf1* gene, significant positive selection was also detected at the nonsyn sites of these genes. A similar phenomenon was also observed in the mitochondrial genome in which 12 of the 38 genes are identified to have undergone positive selection, with each of which possessing several selected nonsyn sites (Table 2). Functions of the 12 genes under selection are related to diverse pathways, including ribosomal protein, succinate dehydrogenase, maturases and ATP synthase.

**Table 2:** Positive selected sites detected in the cytoplasmic genome of the genus *Panax*.

**Genome**	**Gene name**	**M2a model**		**M8 model**	
		**Selected sites**	**Pr (w>1)**	**Selected sites**	**Pr (w>1)**
Chloroplast	*rbcL*	328 S	0.973^*^	328 S	0.984^*^
	*clpP*	37 P	0.988^*^	37 P	0.995^**^
		39 A	0.998^**^	39 A	0.999^**^
	*atpF*	183 G	0.946	183 G	0.977^*^
	*ycf1*	552 K	0.946	552 K	0.955^*^
		709 V	0.981^*^	709 V	0.987^*^
		732 T	0.980^*^	732 T	0.987^*^
		741 R	0.984^*^	741 R	0.990^*^
		744 I	0.990^**^	744 I	0.994^**^
		1040 E	0.986^*^	1040 E	0.991^**^
		1505 Q	0.971^*^	1505 Q	0.979^*^
		1688 E	0.980^*^	1688 E	0.987^*^
	*ycf2*	NA	NA	NA	NA
Mitochondrion	*atp6*	27 H	0.965^*^	27 H	0.984^*^
		48 L	0.951^*^	48 L	0.971^*^
		216 L	0.962^*^	216 L	0.983^*^
	*atp8*	26 P	0.984^*^	26 P	0.991^**^
		80 S	1.000^**^	80 S	1.000^**^
	*matR*	256 H	0.944	256 H	0.958^*^
		288 Q	0.991^**^	288 Q	0.994^**^
		412 L	0.957^*^	412 L	0.966^*^
	*nad3*	8 C	0.980^*^	8 C	0.995^**^
		15 L	0.889	15 L	0.958^*^
	*rps4*	217 K	0.975^*^	217 K	0.990^*^
		269 L	0.977^*^	269 L	0.990^**^
	*rps11*	13 S	0.998^**^	13 S	0.999^**^
	*sdh3*	13 L	0.981^*^	13 L	0.993^**^
		38 L	0.904	38 L	0.954^*^
	*sdh4*	96 L	0.957^*^	96 L	0.982^*^
	*ccmB*	7 E	0.987^*^	7 E	0.997^**^
		78 S	0.916	78 S	0.969^*^
		204 S	0.916	204 S	0.969^*^
	*ccmC*	77 F	0.959^*^	77 F	0.983^*^
	*ccmFc*	393 S	0.990^*^	393 S	0.996^**^
	*ccmFn*	NA	NA	NA	NA

*NA, the two genes, ycf2 and ccmFn show significant selection at both M2a and M8 site models, but no sites are identified in the BEB inference of both models*.

* p < 0.05;

** *p < 0.01*.

## Discussion

### Mechanisms underlying the rate heterogeneity in cytoplasmic genomes

Sequence polymorphisms maintained in the cytoplasmic genomes have long been regarded as selectively neutral (Bock et al., 2014). Under the neutrality assumption, variants are expected to be randomly distributed across the chloroplast and mitochondrial genomes. However, genetic evidence from recent studies pointed out that rate heterogeneity of nucleotide substitution is a general feature of the cytoplasmic genome in flowering plants. In particular, the genes showing accelerated rate of nucleotide substitution are widely observed and shared among the flowering plants (Bock et al., 2014). In this study, rate heterogeneity was also observed in the chloroplast and mitochondrial genomes at both overall and individual species levels in the medicinally important genus *Panax*. Specifically, the two chloroplast inverted regions, IRA and IRB, show obviously lower sequence polymorphisms compared to the LSC and SSC regions. Mechanisms underlying the rate heterogeneity can be explained by different mutation rates and selection pressures among loci (locus-specific effects) or species (lineage effects) (Soria-Hernanz et al., 2008). For example, genetic analyses based on 113 grass chloroplast genomes revealed that both locus-specific and lineage effects have contributed to the heterogeneity of sequence polymorphism (Piot et al., 2017). In our case, results from chloroplast genes demonstrate that, while the two most basal *Panax* species possess clearly more variants relative to those that are phylogenetically closer to *P. ginseng*, all species show a similar pattern of variant distribution, suggesting that locus-specific effect might have played a major role in maintaining the variant distribution in chloroplast genome. In contrast, while heterogeneity in sequence polymorphism is also observed among mitochondrial genes, high sequence polymorphisms are found in the species *P. notoginseng* and *P. bipinnatificus* var. *japonicus* instead of the two most basal species, suggesting that both locus-specific and lineage effects have together shaped the variant pattern of *Panax* mitochondrial genome.

Mitochondrial and chloroplast genomes share many common properties, such as a circular chromosome structure, uniparental inheritance and transferring most of their genes to nuclear genome (reviewed in Smith and Keeling, 2015). Parallel evolution of these common features within the same cell suggests that the two cytoplasmic genomes may have shared similar evolutionary trajectories. However, our results revealed that these *Panax* species show obviously distinct variation pattern between chloroplast and mitochondrial genomes, with the latter possessing more variants at total sites but containing less sequence polymorphism at the genic region. These observations can be partially explained by the DNA repair mechanisms which specifically act on the mitochondrial coding regions (Davila et al., 2011; Christensen, 2013, 2014). In fact, similar phenomena were widely found in flowering plants wherein although structural variation and recombination occurred frequently in the mitochondrial genome, relatively lower nucleotide substitution rates were observed at the genic regions (Drouin et al., 2008; Soria-Hernanz et al., 2008; Zhu et al., 2014). In addition, it was proposed that the mitochondrial genome harbors a greater breadth of complexity and more pronounced unique variations than chloroplast genome. For example, nucleotide substitution rates of mitochondrial genes are more variable and often much higher or lower than those of chloroplast (Lynch et al., 2006; Richardson et al., 2013; Smith and Keeling, 2015). Thus, accelerated substitution rate of mitochondrial genome might also be the potential reason that results in the accumulation of high number of variants at the inter-genic regions. Furthermore, gene transfer from chloroplast and nuclear genome to mitochondrial genome occurred frequently in flowering plants, whereas foreign DNA insertions are very rare in the chloroplast genome (Kleine et al., 2009; Smith, 2011). This process confers the mitochondrion a more complex genomic constitution compared to the chloroplast genome. However, we also realized that the inter-genic variants obtained in this study were relied on the assumption of no structural variations across the *Panax* species. While our results revealed high level of genome coverage for these *Panax* accessions, the possibility of false positive of these mitochondrial inter-genetic variants cannot be fully ruled out. Taken together, our study suggests that DNA repair, nucleotide substitution rate and complicated evolutionary process might be the potential mechanisms that lead to the heterogeneity of sequence polymorphism observed in *Panax* cytoplasmic genomes, especially for the mitochondrial genome.

### Positive selection driving the cytoplasmic genome evolution

All *Panax* species have adapted to cool and shaded environments (Liu and Xiao, 1992; Wen, 2001a; Chen et al., 2014, 2016), indicating that these shade-demanding species might have evolved mechanisms to respond to different light conditions. In the case of *P. notoginseng*, for example, increasing in specific leaf area and chlorophyll content are negatively correlated with the growth irradiance level (Chen et al., 2014, 2016). Likewise, cytoplasmic proteins of *P. ginseng* encoded by both cytoplasmic and nuclear genomes are the targets of selection during local adaptation and domestication processes (Li et al., 2017). These attributes together suggest that cytoplasmic genes may have evolved non-neutrally in the *Panax* species. As expected, five and 12 genes were suggested to have undergone positive selection during the evolutionary process of *Panax*. In the case of chloroplast genome, for example, the large subunit of rubisco gene, *rbcL*, is the target of selection in relation to the changes in temperature, drought and carbon dioxide concentration (reviewed in Bock et al., 2014), in particular for those species that have experienced C4 photosynthesis evolution (Galmés et al., 2015; Orr et al., 2016; Piot et al., 2017). Our study also revealed that positive selection might have acted on the *rbcL* gene during the evolutionary process of *Panax*. Of significance, only one syn substitution was found in the three tetraploid species (*P. ginseng, P. japonicas*, and *P. quinquefolius*) that form a clade, suggesting they evolved via a single recent polyploidy event (Wen and Zimmer, 1996; Shi et al., 2015), whereas a total of 14 syn and 13 nonsyn substitutions were identified in the remaining species. Specifically, these non-syn substitutions are clustered in the N-terminal domain and the middle region, which form the active sites for rubisco and interface with the *rbcS* gene (Spreitzer and Salvucci, 2002; Spreitzer et al., 2005). Given that the genus *Panax* has experienced both ancient and recent WGDs (Shi et al., 2015) and the *rbcL* gene plays important roles in the concerted evolution of *rbcS* gene in allopolyploids (Gong et al., 2012, 2014), we propose that cyto-nuclear coordination between the two rubisco genes might have together contributed to the speciation and adaptation of *Panax* species. In addition, the genes *atpF, clpP, ycf1*, and *ycf2* identified in this study are also the targets of positive selection in diverse flowering plants (Erixon and Oxelman, 2008; Parks et al., 2009; Zhong et al., 2009; Sloan et al., 2014). Functions of these genes are related to plant development and cellular metabolism (Drescher et al., 2000; Kuroda and Maliga, 2003), suggesting that these genes are the common targets in the evolutionary process of chloroplast genome.

Compared to the chloroplast genome that transforms the solar energy into carbohydrates and oxygen, mitochondrial genome is mainly responsible for providing energy currency to life processes of all eukaryotic species (Gray et al., 1999; Gray, 2012; Jensen and Leister, 2014). While genome size and structure of mitochondrion vary dramatically among species, gene content is highly conserved (Gray, 2014; Smith and Keeling, 2015; Gualberto and Newton, 2017), possibly due to strong selective constraints acting on the mitochondrial genes (Björkholm et al., 2015). However, genetic surveys of neutrality in mitochondrial genome are far behind the chloroplast genome in flowering plants. In this study, we found that all *Panax* species show high ratio of non-synonymous/synonymous substitution at the overall level in mitochondrion compared to the chloroplast. Specifically, 12 of the 38 (31.6%) protein-coding genes are assumed to under positive selection. Functions of these genes are related to RNA maturases, succinate dehydrogenase, ATP and ribosomal protein synthesis. The ATP synthesis genes (e.g., *atp1* and *atp9*) show accelerated substitution rate in diverse flowering plants (Mower et al., 2007; Bock et al., 2014). Genetic analyses of the gene *atp1* suggested that it was involved in plant development of the Arabidopsis *recG1* line (Wallet et al., 2015). Similarly, succinate dehydrogenases (*sdh3* and *sdh4*) are functional related to photosynthesis, fungal defense, and reactive oxygen species production (Huang and Millar, 2013; Jardim-Messeder et al., 2015). These observations collectively suggest that the mitochondrial genes under positive selection are highly associated with plant development and adaptation in *Panax*. Taken together, our study demonstrates that while most of the sequence polymorphisms are selectively neutral in the two cytoplasmic genomes, positive selection is one of the potential driving forces modulating the nucleotide variation patterns in the *Panax* species. In particular, the mitochondrial genes evolved under obviously stronger selection pressure compared to the chloroplast genes, possibly due to distinct functions and evolutionary trajectories of the two cytoplasmic genomes in the genus *Panax*. These findings shed new insights into the cytoplasmic genome evolution of the plant species.

## Author contributions

L-FL conceived and designed the experiments. L-FL, BL, JW, PJ, F-XS, M-RL, and H-XX interpreted the data and wrote the manuscript. PJ, F-XS, and M-RL performed the experiments and analyzed the data. All authors contributed to and approved the final manuscript.

### Conflict of interest statement

The authors declare that the research was conducted in the absence of any commercial or financial relationships that could be construed as a potential conflict of interest.

## Supplementary material

Figure S1 Average dead depth of the 11 *Panax* accessions in the chloroplast **(A)** and mitochondrial **(B)** genomes.

Figure S2 Bayesian trees of the genus *Panax* based on the total **(A)** and genic **(B)** variants of mitochondrial genome. Numbers on each branch are the posterior probabilities.

Figure S3 Variant distributions of chloroplast genome in the Panax species at the individual level. The color bars on the bottom represent the location of each gene in the chloroplast genome. Orders of these genes are the same as the Figure 2A.

Figure S4 Variant distributions of mitochondrial genome in the Panax species at the individual level. The color bars on the bottom represent the location of each gene in the mitochondrial genome. Orders of these genes are the same as the Figure 2B.

Table S1 Geographic locations of the 11 Panax accessions used in this study.

Table S2 Numbers of synonymous and non-synonymous sites for each gene of the chloroplast genome in Panax.

Table S3 Numbers of synonymous and non-synonymous sites for each gene of the mitochondrial genome in Panax.

## References

[B1] Abbott R. Albach D. Ansell S. Arntzen J. W. Baird S. J. Bierne N.. (2013). Hybridization and speciation. J. Evol. Biol. 26, 229–246. 10.1111/j.1420-9101.2012.02599.x 23323997

[B2] Allen J. F. (2015). Why chloroplasts and mitochondria retain their own genomes and genetic systems: colocation for redox regulation of gene expression. Proc. Natl. Acad. Sci. U.S.A. 112, 10231–10238. 10.1073/pnas.1500012112 26286985 PMC4547249

[B3] Björkholm P. Harish A. Hagström E. Ernst A. M. Andersson S. G. (2015). Mitochondrial genomes are retained by selective constraints on protein targeting. Proc. Natl. Acad. Sci. U.S.A. 112, 10154–10161. 10.1073/pnas.1421372112 26195779 PMC4547212

[B4] Bock D. G. Andrew R. L. Rieseberg L. H. (2014). On the adaptive value of cytoplasmic genomes in plants. Mol. Ecol. 23, 4899–4911. 10.1111/mec.12920 25223488

[B5] Chen J. W. Kuang S. B. Long G. Q. Meng Z. G. Li L. G. Chen Z. J. (2014). Steady-state and dynamic photosynthetic performance and nitrogen partitioning in the shade-demanding plant *Panax notoginseng* under different levels of growth irradiance. Acta Physiol. Plant. 36, 2409–2420. 10.1007/s11738-014-1614-9

[B6] Chen J. W. Kuang S. B. Long G. Q. Yang S. C. Meng Z. G. Li L. G. (2016). Photosynthesis, light energy partitioning, and photoprotection in the shade-demanding species *Panax notoginseng* under high and low level of growth irradiance. Funct. Plant Biol. 43, 479–491. 10.1071/FP15283 32480478

[B7] Chen W. Kui L. Zhang G. Zhu S. Zhang J. Wang X.. (2017a). Whole-genome sequencing and analysis of the Chinese herbal plant *Panax notoginseng*. Mol. Plant. 10, 899–902. 10.1016/j.molp.2017.02.010 28315474

[B8] Chen Z. Zhao N. Li S. Grover C. E. Nie H. Wendel J. F. (2017b). Plant mitochondrial genome evolution and cytoplasmic male sterility. Crit. Rev. Plant. Sci. 36, 55–69. 10.1080/07352689.2017.1327762

[B9] Choi H. I. Kim N. H. Lee J. Choi B. S. Do Kim K. Park J. Y. (2013). Evolutionary relationship of Panax ginseng and P. *quinquefolius* inferred from sequencing and comparative analysis of expressed sequence tags. Genet. Resour. Crop. Evol. 60, 1377–1387. 10.1007/s10722-012-9926-3

[B10] Choi H. I. Waminal N. E. Park H. M. Kim N. H. Choi B. S. Park M. (2014). Major repeat components covering one-third of the ginseng (*Panax ginseng* CA Meyer) genome and evidence for allotetraploidy. Plant J. 77, 906–916. 10.1111/tpj.12441 24456463

[B11] Choi H. K. Wen J. (2000). A phylogenetic analysis of *Panax* (Araliaceae): integrating cpDNA restriction site and nuclear rDNA ITS sequence data. Plant Syst. Evol. 224, 109–120. 10.1007/BF00985269

[B12] Christensen A. C. (2013). Plant mitochondrial genome evolution can be explained by DNA repair mechanisms. Genome Biol. Evol. 5, 1079–1086. 10.1093/gbe/evt069 23645599 PMC3698917

[B13] Christensen A. C. (2014). Genes and junk in plant mitochondria-repair mechanisms and selection. Genome Biol. Evol. 6, 1448–1453. 10.1093/gbe/evu115 24904012 PMC4079193

[B14] Danecek P. Auton A. Abecasis G. Albers C. A. Banks E. DePristo M. A.. (2011). The variant call format and VCFtools. Bioinformatics 27, 2156–2158. 10.1093/bioinformatics/btr330 21653522 PMC3137218

[B15] Daniell H. Lin C. S. Yu M. Chang W. J. (2016). Chloroplast genomes: diversity, evolution, and applications in genetic engineering. Genome Biol. 17:134. 10.1186/s13059-016-1004-2 27339192 PMC4918201

[B16] Darriba D. Taboada G. L. Doallo R. Posada D. (2012). jModelTest 2: more models, new heuristics and parallel computing. Nat. Methods 9, 772–772. 10.1038/nmeth.2109 22847109 PMC4594756

[B17] Davila J. I. Arrieta-Montiel M. P. Wamboldt Y. Cao J. Hagmann J. Shedge V.. (2011). Double-strand break repair processes drive evolution of the mitochondrial genome in Arabidopsis. BMC Biol. 9:64. 10.1186/1741-7007-9-64 21951689 PMC3193812

[B18] De Bruijn S. Angenent G. C. Kaufmann K. (2012). Plant “evo-devo”goes genomic: from candidate genes to regulatory networks. Trends Plant Sci. 17, 441–447. 10.1016/j.tplants.2012.05.002 22698378

[B19] Drescher A. Ruf S. Calsa T. Carrer H. Bock R. (2000). The two largest chloroplast genome-encoded open reading frames of higher plants are essential genes. Plant J. 22, 97–104. 10.1046/j.1365-313x.2000.00722.x 10792825

[B20] Drouin G. Daoud H. Xia J. (2008). Relative rates of synonymous substitutions in the mitochondrial, chloroplast and nuclear genomes of seed plants. Mol. Phylogenet. Evol. 49, 827–831. 10.1016/j.ympev.2008.09.009 18838124

[B21] Erixon P. Oxelman B. (2008). Whole-gene positive selection, elevated synonymous substitution rates, duplication, and indel evolution of the chloroplast *clpP1* gene. PLoS ONE 3:e1386. 10.1371/journal.pone.0001386 18167545 PMC2148103

[B22] Fernández-Mazuecos M. Glover B. J. (2017). The evo-devo of plant speciation. Nat. Ecol. Evol. 1:0110. 10.1038/s41559-017-0110 28812669

[B23] Galmés J. Kapralov M. Copolovici L. Hermida-Carrera C. Niinemets Ü. (2015). Temperature responses of the Rubisco maximum carboxylase activity across domains of life: phylogenetic signals, trade-offs, and importance for carbon gain. Photosyn. Res. 123, 183–201. 10.1007/s11120-014-0067-8 25515770

[B24] Gong L. Olson M. Wendel J. F. (2014). Cytonuclear evolution of rubisco in four allopolyploid lineages. Mol. Biol. Evol. 31, 2624–2636. 10.1093/molbev/msu207 25015644 PMC4166922

[B25] Gong L. Salmon A. Yoo M. J. Grupp K. K. Wang Z. Paterson A. H.. (2012). The cytonuclear dimension of allopolyploid evolution: an example from cotton using rubisco. Mol. Biol. Evol. 29, 3023–3036. 10.1093/molbev/mss110 22490824

[B26] Gray M. W. (1992). The endosymbiont hypothesis revisited. Int. Rev. Cytol. 141, 233–357. 10.1016/S0074-7696(08)62068-9 1452433

[B27] Gray M. W. (2012). Mitochondrial evolution. CSH Perspect. Biol. 4:a011403. 10.1101/cshperspect.a011403 22952398 PMC3428767

[B28] Gray M. W. (2014). The pre-endosymbiont hypothesis: a new perspective on the origin and evolution of mitochondria. CSH Perspect. Biol. 6:a016097. 10.1101/cshperspect.a016097 24591518 PMC3949359

[B29] Gray M. W. Burger G. Lang B. F. (1999). Mitochondrial evolution. Science 283, 1476–1481. 10.1126/science.283.5407.1476 10066161

[B30] Greiner S. Bock R. (2013). Tuning a ménage à trois: co-evolution and co-adaptation of nuclear and organellar genomes in plants. Bioessays 35, 354–365. 10.1002/bies.201200137 23361615

[B31] Gualberto J. M. Newton K. J. (2017). Plant mitochondrial genomes: dynamics and mechanisms of mutation. Annu. Rev. Plant Biol. 68, 225–252. 10.1146/annurev-arplant-043015-112232 28226235

[B32] Guisinger M. M. Kuehl J. V. Boore J. L. Jansen R. K. (2008). Genome-wide analyses of Geraniaceae plastid DNA reveal unprecedented patterns of increased nucleotide substitutions. Proc. Natl. Acad. Sci. U.S.A. 105, 18424–18429. 10.1073/pnas.0806759105 19011103 PMC2587588

[B33] Han Z. J. Li W. Liu Y. Gao L. Z. (2016). The complete chloroplast genome of North American ginseng, *Panax quinquefolius*. Mitochondrial DNA 27, 3496–3497. 10.3109/19401736.2015.1066365 27158867

[B34] Hjort K. Goldberg A. V. Tsaousis A. D. Hirt R. P. Embley T. M. (2010). Diversity and reductive evolution of mitochondria among microbial eukaryotes. Philos. Trans. R. Soc. Lond. B Biol. Sci. 365, 713–727. 10.1098/rstb.2009.0224 20124340 PMC2817227

[B35] Huang S. Millar A. H. (2013). Succinate dehydrogenase: the complex roles of a simple enzyme. Curr. Opin. Plant Biol. 16, 344–349. 10.1016/j.pbi.2013.02.007 23453781

[B36] Jardim-Messeder D. Caverzan A. Rauber R. Souza Ferreira E. Margis-Pinheiro M. Galina A. (2015). Succinate dehydrogenase (mitochondrial complex II) is a source of reactive oxygen species in plants and regulates development and stress responses. New Phytol. 208, 776–789. 10.1111/nph.13515 26082998

[B37] Jensen P. E. Leister D. (2014). Chloroplast evolution, structure and functions. F1000prime Rep. 6:40. 10.12703/P6-40 24991417 PMC4075315

[B38] Jiang X. Yang C. Baosheng L. Shuiming X. Qinggang Y. Rui B. (2017). *Panax ginseng* genome examination for ginsenoside biosynthesis. GigaScience 6, 1–15. 10.1093/gigascience/gix093 PMC5710592 29048480

[B39] Kim N.-H. Choi H.-I. Kim K. H. Jang W. Yang T.-J. (2014). Evidence of genome duplication revealed by sequence analysis of multi-loci expressed sequence tag-simple sequence repeat bands in *Panax ginseng* Meyer. J. Gins. Res. 38, 130–135. 10.1016/j.jgr.2013.12.005 24748837 PMC3986581

[B40] Kleine T. Maier U. G. Leister D. (2009). DNA transfer from organelles to the nucleus: the idiosyncratic genetics of endosymbiosis. Annu. Rev. Plant Biol. 60, 115–138. 10.1146/annurev.arplant.043008.092119 19014347

[B41] Kuroda H. Maliga P. (2003). The plastid clpP1 protease gene is essential for plant development. Nature 425, 86–89. 10.1038/nature01909 12955146

[B42] Lee C. Wen J. (2004). Phylogeny of *Panax* using chloroplast *trnC*–*trnD* intergenic region and the utility of *trnC*–*trnD* in interspecific studies of plants. Mol. Phylogenet. Evol. 31, 894–903. 10.1016/j.ympev.2003.10.009 15120387

[B43] Li H. Durbin R. (2010). Fast and accurate long-read alignment with Burrows–Wheeler transform. Bioinformatics 26, 589–595. 10.1093/bioinformatics/btp698 20080505 PMC2828108

[B44] Li H. Handsaker B. Wysoker A. Fennell T. Ruan J. Homer N.. (2009). The sequence alignment/map format and SAMtools. Bioinformatics 25, 2078–2079. 10.1093/bioinformatics/btp352 19505943 PMC2723002

[B45] Li M. R. Shi F. X. Li Y. L. Jiang P. Jiao L. Liu B.. (2017). Genome-wide variation patterns uncover the origin and selection in cultivated ginseng (*Panax ginseng* Meyer). Genome Biol. Evol. 9, 2159–2169. 10.1093/gbe/evx160 28922794 PMC5737880

[B46] Li M. R. Shi F. X. Zhou Y. X. Li Y. L. Wang X. F. Zhang C.. (2015). Genetic and epigenetic diversities shed light on domestication of cultivated ginseng (*Panax ginseng*). Mol. Plant 8, 1612–1622. 10.1016/j.molp.2015.07.011 26278367

[B47] Li R. Ma P.-F. Wen J. Yi T.-S. (2013). Complete sequencing of five Araliaceae chloroplast genomes and the phylogenetic implications. PLoS ONE 8:e78568. 10.1371/journal.pone.0078568 24205264 PMC3799623

[B48] Li R. Wen J. (2016). Phylogeny and diversification of Chinese Araliaceae based on nuclear and plastid DNA sequence data. J. Syst. Evol. 54, 453–467. 10.1111/jse.12196

[B49] Liu C. X. Xiao P. G. (1992). Recent advances on ginseng research in China. J. Ethnopharmacol. 36, 27–38. 10.1016/0378-8741(92)90057-X 1501490

[B50] Lynch M. Koskella B. Schaack S. (2006). Mutation pressure and the evolution of organelle genomic architecture. Science 311, 1727–1730. 10.1126/science.1118884 16556832

[B51] Mower J. P. Touzet P. Gummow J. S. Delph L. F. Palmer J. D. (2007). Extensive variation in synonymous substitution rates in mitochondrial genes of seed plants. BMC Evol. Biol. 7:135. 10.1186/1471-2148-7-135 17688696 PMC1973135

[B52] Orr D. Alcântara A. Kapralov M. V. Andralojc J. Carmo-Silva E. Parry M. A. J. (2016). Surveying Rubisco diversity and temperature response to improve crop photosynthetic efficiency. Plant Physiol. 172, 707–717. 10.1104/pp.16.00750 27342312 PMC5047088

[B53] Parks M. Cronn R. Liston A. (2009). Increasing phylogenetic resolution at low taxonomic levels using massively parallel sequencing of chloroplast genomes. BMC Biol. 7:84. 10.1186/1741-7007-7-84 19954512 PMC2793254

[B54] Patel R. K. Jain M. (2012). NGS QC toolkit: a platform for quality control of next-generation sequencing data. PLoS ONE 7:e30619 10.1371/journal.pone.0030619 22312429 PMC3270013

[B55] Piot A. Hackel J. Christin P.-A. Besnard G. (2017). One-third of the plastid genes evolved under positive selection in PACMAD grasses. Planta 247, 255–266. 10.1007/s00425-017-2781-x 28956160

[B56] Rambaut A. (2009). FigTree, version 1.3. 1. Computer Program Distributed by the Author. Available Online at: http://tree.bio.ed.ac.uk/software/figtree/ (Accessed January 4, 2011).

[B57] Richardson A. O. Rice D. W. Young G. J. Alverson A. J. Palmer J. D. (2013). The “fossilized” mitochondrial genome of Liriodendron tulipifera: ancestral gene content and order, ancestral editing sites, and extraordinarily low mutation rate. BMC Biol. 11:29. 10.1186/1741-7007-11-29 23587068 PMC3646698

[B58] Ronquist F. Teslenko M. Van Der Mark P. Ayres D. L. Darling A. Höhna S.. (2012). MrBayes 3.2: efficient Bayesian phylogenetic inference and model choice across a large model space. Syst. Biol. 61, 539–542. 10.1093/sysbio/sys029 22357727 PMC3329765

[B59] Roux F. Mary-Huard T. Barillot E. Wenes E. Botran L. Durand S.. (2016). Cytonuclear interactions affect adaptive traits of the annual plant Arabidopsis thaliana in the field. Proc. Natl. Acad. Sci. U.S.A. 113, 3687–3692. 10.1073/pnas.1520687113 26979961 PMC4822599

[B60] Schmieder R. Edwards R. (2011). Quality control and preprocessing of metagenomic datasets. Bioinformatics 27, 863–864. 10.1093/bioinformatics/btr026 21278185 PMC3051327

[B61] Sharbrough J. Conover J. L. Tate J. A. Wendel J. F. Sloan D. B. (2017). Cytonuclear responses to genome doubling. Am. J. Bot. 104, 1277–1280. 10.3732/ajb.1700293 29885242

[B62] Sherman-Broyles S. Bombarely A. Doyle J. (2017). Characterizing the allopolyploid species among the wild relatives of soybean: utility of reduced representation genotyping methodologies. J. Syst. Evol. 55, 365–376. 10.1111/jse.12268

[B63] Shi F. X. Li M. R. Li Y. L. Jiang P. Zhang C. Pan Y. Z.. (2015). The impacts of polyploidy, geographic and ecological isolations on the diversification of *Panax* (Araliaceae). BMC Plant Biol. 15:297. 10.1186/s12870-015-0669-0 26690782 PMC4687065

[B64] Skippington E. Barkman T. J. Rice D. W. Palmer J. D. (2015). Miniaturized mitogenome of the parasitic plant *Viscum scurruloideum* is extremely divergent and dynamic and has lost all nad genes. Proc. Natl. Acad. Sci. U.S.A. 112, E3515–E3524. 10.1073/pnas.1504491112 26100885 PMC4500244

[B65] Sloan D. B. Müller K. McCauley D. E. Taylor D. R. Štorchová H. (2012). Intraspecific variation in mitochondrial genome sequence, structure, and gene content in Silene vulgaris, an angiosperm with pervasive cytoplasmic male sterility. New Phytol. 196, 1228–1239. 10.1111/j.1469-8137.2012.04340.x 23009072

[B66] Sloan D. B. Triant D. A. Forrester N. J. Bergner L. M. Wu M. Taylor D. R. (2014). A recurring syndrome of accelerated plastid genome evolution in the angiosperm tribe *Sileneae* (Caryophyllaceae). Mol. Phylogenet. Evol. 72, 82–89. 10.1016/j.ympev.2013.12.004 24373909

[B67] Sloan D. B. Triant D. A. Wu M. Taylor D. R. (2013). Cytonuclear interactions and relaxed selection accelerate sequence evolution in organelle ribosomes. Mol. Biol. Evol. 31, 673–682. 10.1093/molbev/mst259 24336923

[B68] Smith D. R. (2011). Extending the limited transfer window hypothesis to inter-organelle DNA migration. Genome Biol. Evol. 3, 743–748. 10.1093/gbe/evr068 21803764 PMC3163470

[B69] Smith D. R. Keeling P. J. (2015). Mitochondrial and plastid genome architecture: reoccurring themes, but significant differences at the extremes. Proc. Natl. Acad. Sci. U.S.A. 112, 10177–10184. 10.1073/pnas.1422049112 25814499 PMC4547224

[B70] Soltis P. S. Marchant D. B. Van de Peer Y. Soltis D. E. (2015). Polyploidy and genome evolution in plants. Curr. Opin. Genet. Dev. 35, 119–125. 10.1016/j.gde.2015.11.003 26656231

[B71] Soria-Hernanz D. F. Braverman J. M. Hamilton M. B. (2008). Parallel rate heterogeneity in chloroplast and mitochondrial genomes of Brazil nut trees (Lecythidaceae) is consistent with lineage effects. Mol. Biol. Evol. 25, 1282–1296. 10.1093/molbev/msn074 18385219

[B72] Spoelhof J. P. Soltis P. S. Soltis D. E. (2017). Pure polyploidy: closing the gaps in autopolyploid research. J. Syst. Evol. 55, 340–352. 10.1111/jse.12253

[B73] Spreitzer R. J. Peddi S. R. Satagopan S. (2005). Phylogenetic engineering at an interface between large and small subunits imparts land-plant kinetic properties to algal Rubisco. Proc. Natl. Acad. Sci. U.S.A. 102, 17225–17230. 10.1073/pnas.0508042102 16282373 PMC1287997

[B74] Spreitzer R. J. Salvucci M. E. (2002). Rubisco: structure, regulatory interactions, and possibilities for a better enzyme. Annu. Rev. Plant Biol. 53, 449–75. 10.1146/annurev.arplant.53.100301.135233 12221984

[B75] Turmel M. Otis C. Lemieux C. (2002). The chloroplast and mitochondrial genome sequences of the charophyte Chaetosphaeridium globosum: insights into the timing of the events that restructured organelle DNAs within the green algal lineage that led to land plants. Proc. Natl. Acad. Sci. U.S.A. 99, 11275–11280. 10.1073/pnas.162203299 12161560 PMC123247

[B76] Wallet C. Le Ret M. Bergdoll M. Bichara M. Dietrich A. Gualberto J. M. (2015). The RECG1 DNA translocase is a key factor in recombination surveillance, repair, and segregation of the mitochondrial DNA in *Arabidopsis*. Plant Cell 27, 2907–2925. 10.1105/tpc.15.00680 26462909 PMC4682331

[B77] Wen J. (2001a). Species diversity, nomenclature, phylogeny, biogeography, and classification of the ginseng genus (*Panax*, L., Araliaceae), in Utilization of Biotechnological, Genetic and Cultural Approaches for North American and Asian Ginseng Improvement. Proceedings of the International Ginseng Workshop, ed Punja Z. K. (Vancouver, BC: Simon Fraser University Press), 67–88.

[B78] Wen J. (2001b). Evolution of the *Aralia*-*Panax* complex (Araliaceae) as inferred from nuclear ribosomal ITS sequences. Edinb. J. Bot. 58, 183–200. 10.1017/S0960428601000610

[B79] Wen J. (2011). Systematics and biogeography of Aralia, L. (*Araliaceae*): revision of Aralia sects. Aralia, Humiles, Nanae, and Sciadodendron. Contr. U.S. Natl. Herb. 57, 1–172.

[B80] Wen J. Zimmer E. A. (1996). Phylogeny and biogeography of *Panax*, L.(the ginseng genus, Araliaceae): inferences from ITS sequences of nuclear ribosomal DNA. Mol. Phylogenet. Evol. 6, 167–177. 10.1006/mpev.1996.0069 8899721

[B81] Wendel J. F. Jackson S. A. Meyers B. C. Wing R. A. (2016). Evolution of plant genome architecture. Genome Biol. 17:37. 10.1186/s13059-016-0908-1 26926526 PMC4772531

[B82] Weng M. L. Ruhlman T. A. Gibby M. Jansen R. K. (2012). Phylogeny, rate variation, and genome size evolution of *Pelargonium* (Geraniaceae). Mol. Phylogenet. Evol. 64, 654–670. 10.1016/j.ympev.2012.05.026 22677167

[B83] Wicke S. Schneeweiss G. M. Müller K. F. Quandt D. (2011). The evolution of the plastid chromosome in land plants: gene content, gene order, gene function. Plant Mol. Biol. 76, 273–297. 10.1007/s11103-011-9762-4 21424877 PMC3104136

[B84] Yagi Y. Shiina T. (2014). Recent advances in the study of chloroplast gene expression and its evolution. Front. Plant. Sci. 5:61. 10.3389/fpls.2014.00061 24611069 PMC3933795

[B85] Yang Z. (2007). PAML 4: phylogenetic analysis by maximum likelihood. Mol. Biol. Evol. 24, 1586–1591. 10.1093/molbev/msm088 17483113

[B86] Yang Z. Wong W. S. Nielsen R. (2005). Bayes empirical Bayes inference of amino acid sites under positive selection. Mol. Biol. Evol. 22, 1107–1118. 10.1093/molbev/msi097 15689528

[B87] Zhang D. Li W. Gao C. Liu Y. Gao L. Z. (2016). The complete plastid genome sequence of *Panax notoginseng*, a famous traditional Chinese medicinal plant of the family Araliaceae. Mitochondrial DNA 27, 3438–3439. 10.3109/19401736.2015.1063131 26365031

[B88] Zhang D. Li W. Xia E. H. Zhang Q. J. Liu Y. Zhang Y.. (2017). The medicinal herb *Panax notoginseng* genome provides insights into ginsenoside biosynthesis and genome evolution. Mol. Plant. 10, 903–907. 10.1016/j.molp.2017.02.011 28315473

[B89] Zhao Y. Yin J. Guo H. Zhang Y. Xiao W. Sun C.. (2015). The complete chloroplast genome provides insight into the evolution and polymorphism of *Panax ginseng*. Front. Plant Sci. 5:696. 10.3389/fpls.2014.00696 25642231 PMC4294130

[B90] Zhong B. Yonezawa T. Zhong Y. Hasegawa M. (2009). Episodic evolution and adaptation of chloroplast genomes in ancestral grasses. PLoS ONE 4:e5297. 10.1371/journal.pone.0005297 19390686 PMC2669172

[B91] Zhu A. Guo W. Jain K. Mower J. P. (2014). Unprecedented heterogeneity in the synonymous substitution rate within a plant genome. Mol. Biol. Evol. 31, 1228–1236. 10.1093/molbev/msu079 24557444

[B92] Zimorski V. Ku C. Martin W. F. Gould S. B. (2014). Endosymbiotic theory for organelle origins. Curr. Opin. Microbiol. 22, 38–48. 10.1016/j.mib.2014.09.008 25306530

[B93] Zuo Y. J. Chen Z. J. Kondo K. Funamoto T. Wen J. Zhou S. L. (2011). DNA barcoding of *Panax* species. Planta Med. 77, 182–187. 10.1055/s-0030-1250166 20803416

[B94] Zuo Y. J. Wen J. Ma J. S. Zhou S. L. (2015). Evolutionary radiation of the *Panax bipinnatifidus* species complex (Araliaceae) in the Sino-Himalayan region of eastern Asia as inferred from AFLP analysis. J. Syst. Evol. 53, 210–220. 10.1111/jse.12119

[B95] Zuo Y. J. Wen J. Zhou S. L. (2017). Intercontinental and intracontinental biogeography of the eastern Asian–eastern North American disjunct *Panax* (the ginseng genus, Araliaceae), emphasizing its diversification processes in eastern Asia. Mol. Phylogenet. Evol. 117, 60–74. 10.1016/j.ympev.2017.06.016 28743642

