# Supplementary figures and images for: Positive Selection Driving Cytoplasmic Genome Evolution of the Medicinally Important Ginseng Plant Genus Panax
Source: Front Plant Sci. 2018 Apr 4;9:359. doi: 10.3389/fpls.2018.00359 (PMC5893753; doi:10.3389/fpls.2018.00359)

### chloroplast genome

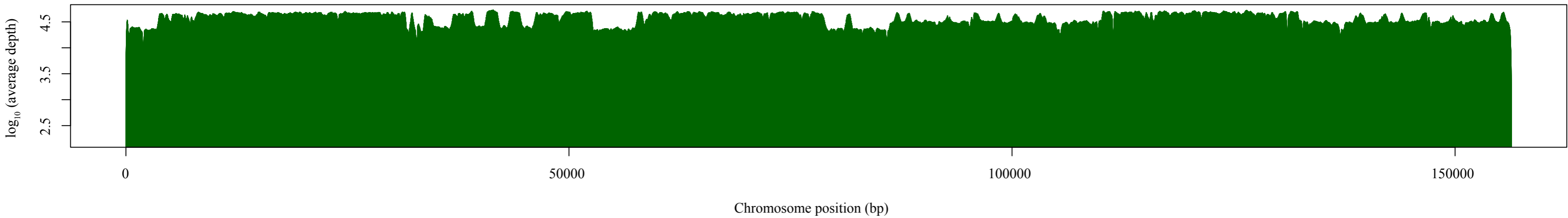

### mitochondrion genome

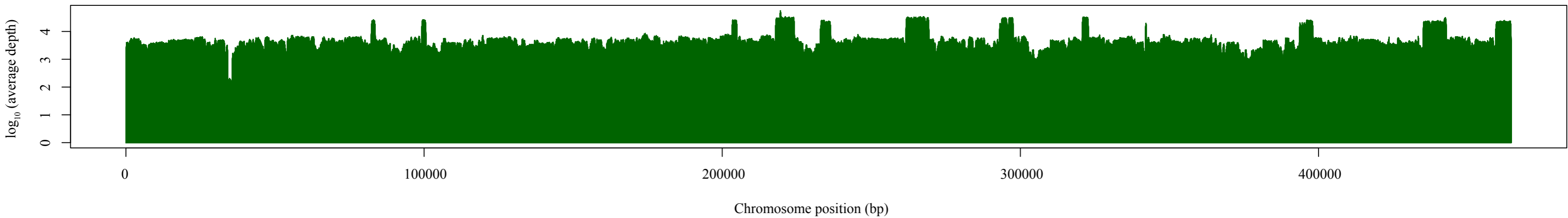

Supplement: Figure S1 — Average dead depth of the 11 Panax accessions in the chloroplast (A) and mitochondrial (B) genomes. [file Image1.PDF]

A

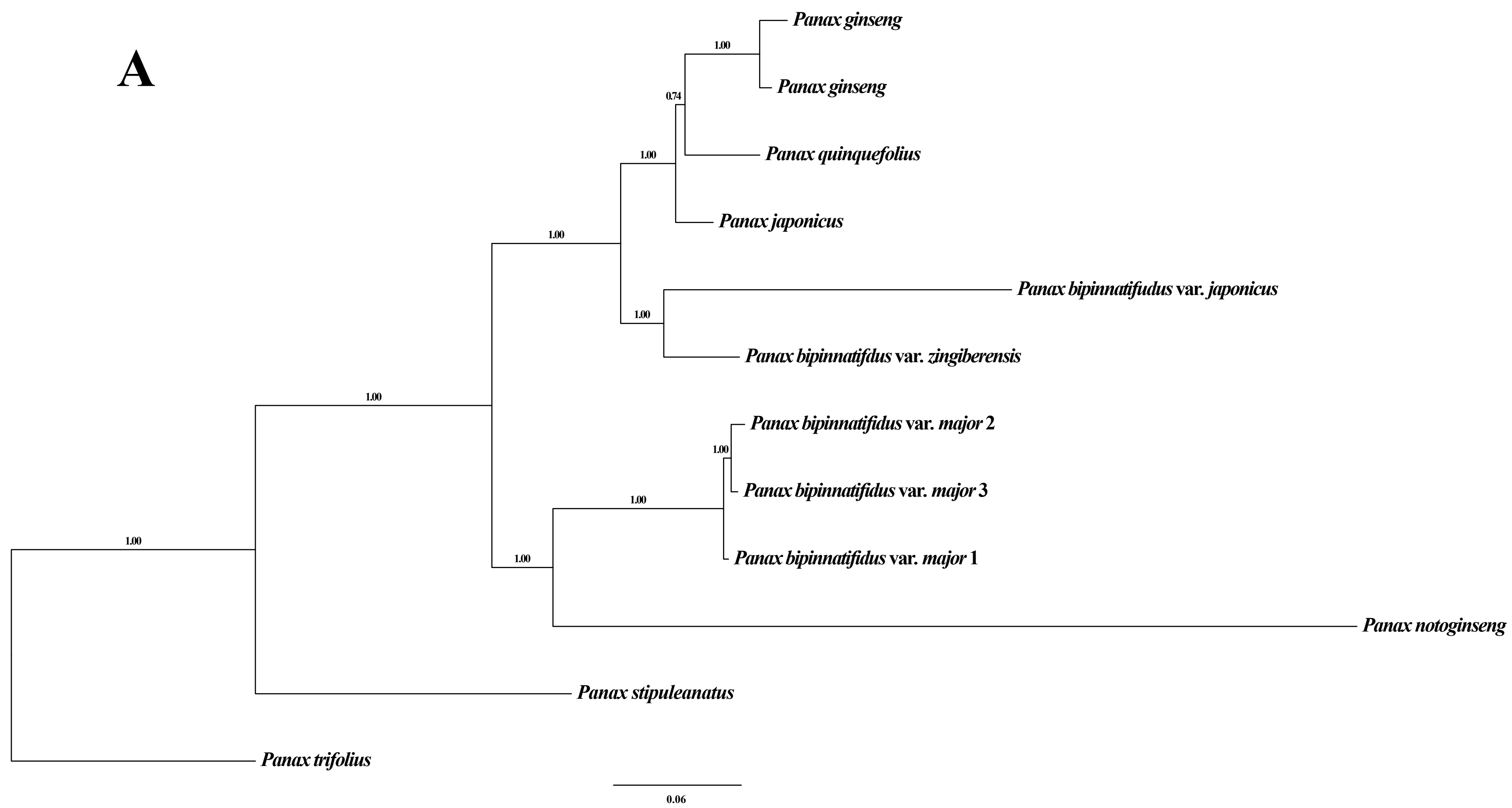

B

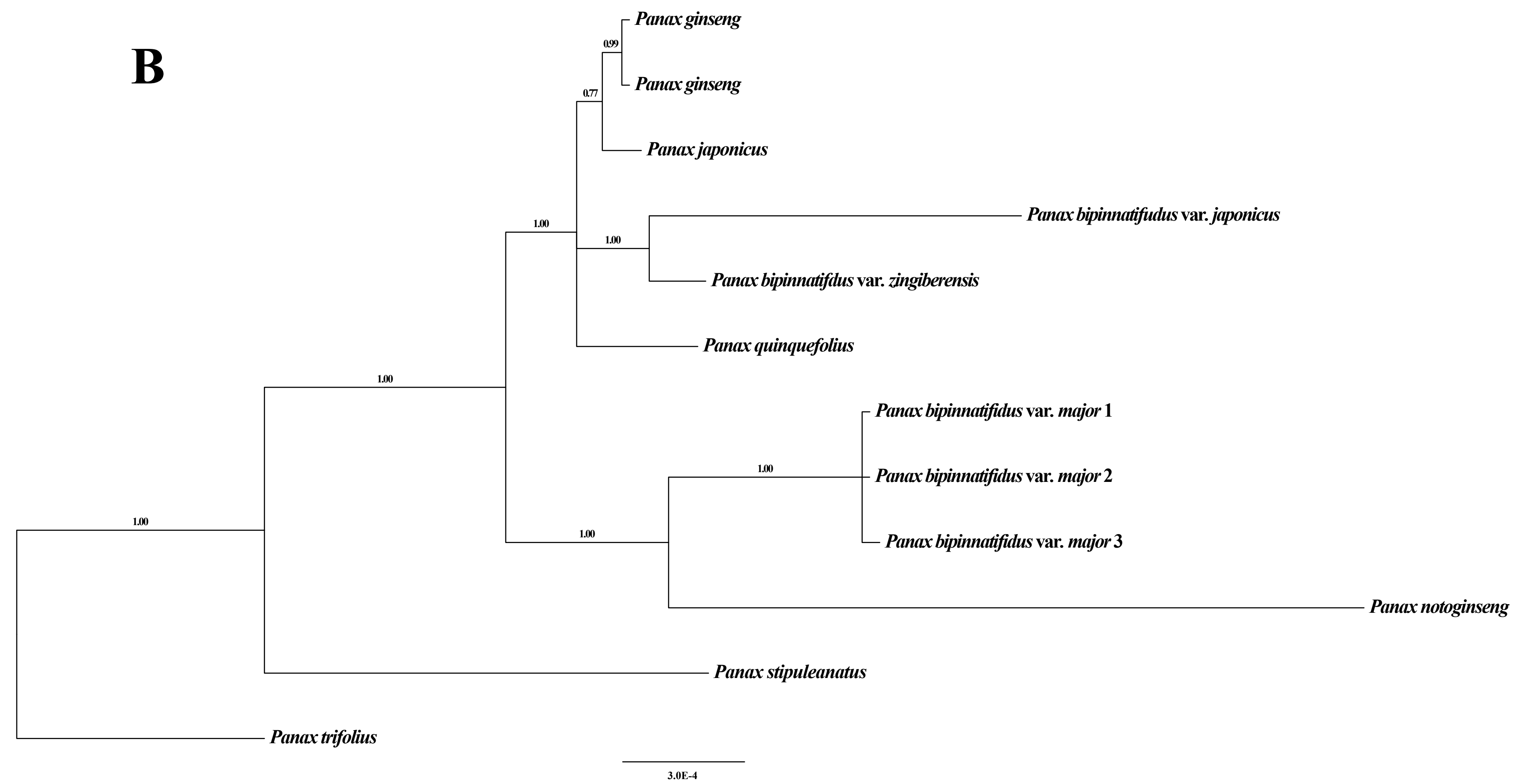

Supplement: Figure S2 — Bayesian trees of the genus Panax based on the total (A) and genic (B) variants of mitochondrial genome. Numbers on each branch are the posterior probabilities. [file Image2.PDF]
