# Supplementary material for: Positive Selection Driving Cytoplasmic Genome Evolution of the Medicinally Important Ginseng Plant Genus Panax
Source: Front Plant Sci. 2018 Apr 4;9:359. doi: 10.3389/fpls.2018.00359 (PMC5893753; doi:10.3389/fpls.2018.00359)
Supplement: Table S1 — Geographic locations of the 11 Panax accessions used in this study. [file Table1.DOCX]

Table S1. Geographic locations of the 11 *Panax* accessions used in this study.

| Species name | Geographic location | Coordination |
| --- | --- | --- |
| *Panax ginseng* | South Korea* | NA |
| *Panax quinquefolius* | Ji’an, Jilin, China | 125.929062, 41.548358 |
| *Panax japonicus* | Japan^#^ | NA |
| *Panax bipinnatifudus* var*. major* 1 | Kunming, Yunnan, China | 102.787081, 25.085984 |
| *Panax bipinnatifudus* var*. major* 2 | Kunming, Yunnan, China | 102.787081, 25.085984 |
| *Panax bipinnatifudus* var*. major* 3 | Kunming, Yunnan, China | 102.787081, 25.085984 |
| *Panax bipinnatifudus* var*. japonicus* | Techong, Yunnan, China | 98.555726, 25.16456 |
| *Panax bipinnatifudus* var*. zingiberensis* | Kunming, Yunnan, China | 102.787081, 25.085984 |
| *Panax notoginseng* | Kunming, Yunnan, China | 102.787081, 25.085984 |
| *Panax stipuleanatus* | Kunming, Yunnan, China | 102.787081, 25.085984 |
| *Panax trifolius* | USA^#^ | NA |

Note: *, data was downloaded from GenBank according to Choi *et al.,* 2014; #, materials were obtained from commercial market; NA, no information is

available.
