# Supplementary material for: Positive Selection Driving Cytoplasmic Genome Evolution of the Medicinally Important Ginseng Plant Genus Panax
Source: Front Plant Sci. 2018 Apr 4;9:359. doi: 10.3389/fpls.2018.00359 (PMC5893753; doi:10.3389/fpls.2018.00359)
Supplement: Figure S3 — Variant distributions of chloroplast genome in the Panax species at the individual level. The color bars on the bottom represent the location of each gene in the chloroplast genome. Orders of these genes are the same as the Figure 2A. [file Image3.PDF]

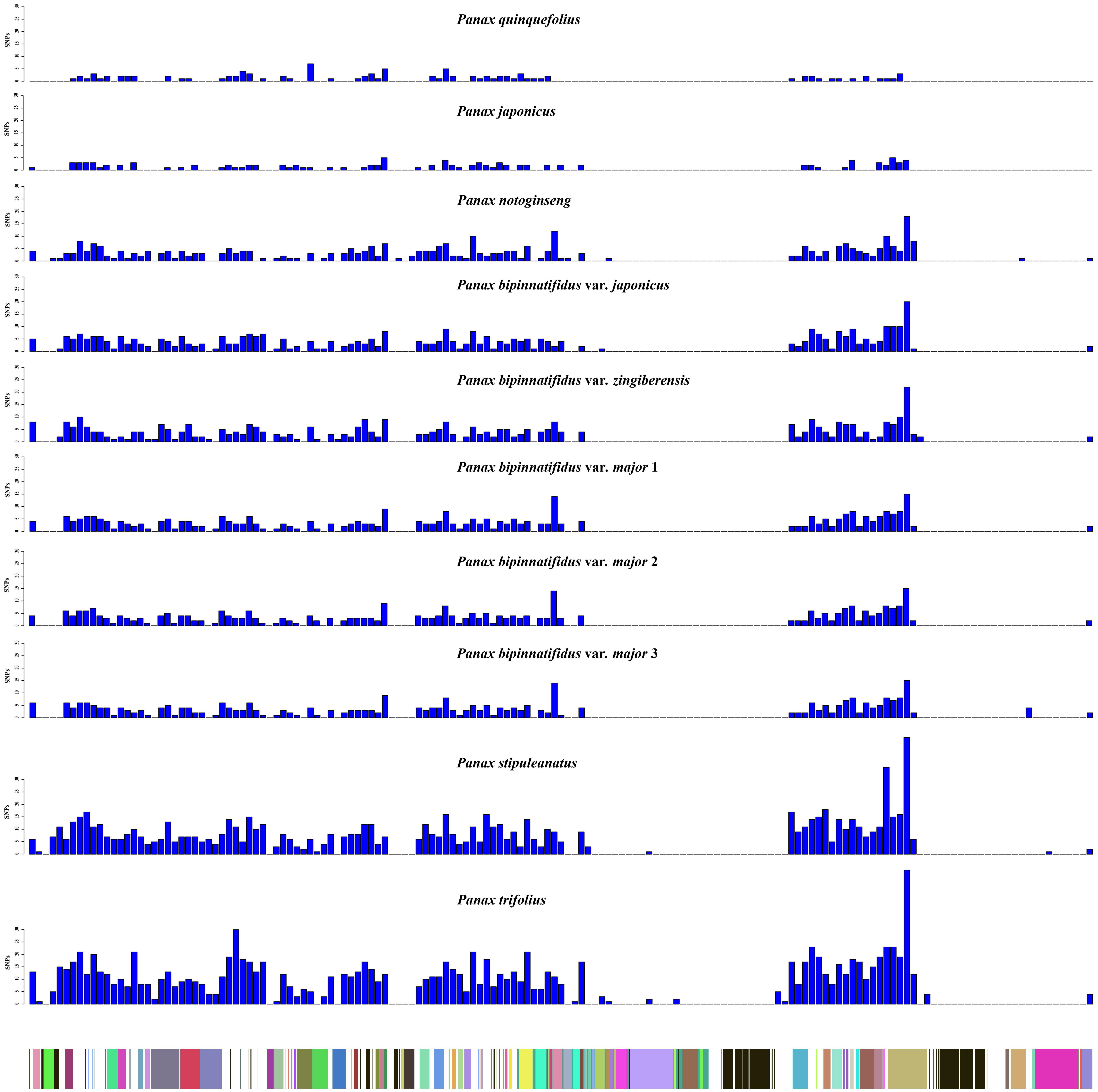
Panax quinquefolius
Panax japonicus
Panax notoginseng
Panax bipinnatifidus var. japonicus
Panax bipinnatifidus var. zingiberensis
Panax bipinnatifidus var. major 1
Panax bipinnatifidus var. major 2
Panax bipinnatifidus var. major 3
Panax stipuleanatus
Panax trifolius
SNPs
